# L-VTP: Long-Term Vessel Trajectory Prediction Based on Multi-Source Data Analysis †

**DOI:** 10.3390/s19204365

**Published:** 2019-10-09

**Authors:** Chao Liu, Shuai Guo, Yuan Feng, Feng Hong, Haiguang Huang, Zhongwen Guo

**Affiliations:** 1 Department of Information Science and Engineering, Ocean University of China, Qingdao 266000, China; liuchao@ouc.edu.cn (C.L.); fengyuan@ouc.edu.cn (Y.F.); hongfeng@ouc.edu.cn (F.H.); guozhw@ouc.edu.cn (Z.G.); 2 Department of Science, Qingdao University of Technology, Qingdao 266000, China; 3 Department of Information Technology, Wenzhou Vocational College of Science and Technology, Wenzhou 325006, China; haiguang2000@wzvcst.edu.cn

**Keywords:** vessel trajectory prediction, entropy analysis, marine IoT, ocean MDTN, K-order Markov chain

## Abstract

With the rapid development of marine IoT (Internet of Things), ocean MDTN (Mobile Delay Tolerant Network) has become a research hot spot. Long-term trajectory prediction is a key issue in MDTN. There are no long-term fine-grained trajectory prediction methods proposed for ocean vessels because a vessel’s mobility pattern lacks map topology support and can be easily influenced by the fish moratorium, sunshine duration, etc. A traditional on-land trajectory prediction algorithm cannot be directly utilized in this field because trajectory characteristics of ocean vessels are far different from that on land. To address the problem above, we propose a novel long-term trajectory prediction algorithm for ocean vessels, called L-VTP, by utilizing multiple sailing related parameters and K-order multivariate Markov Chain. L-VTP utilizes multiple sailing related parameters to build multiple state-transition matrices for trajectory prediction based on quantitative uncertainty analysis of trajectories. Trajectories’ sparsity of ocean vessels results in a critical state missing problem of a high-order state-transition matrix. L-VTP automatically traverses other matrices in a specific sequence in terms of quantitative uncertainty results to overcome this problem. Furthermore, the different mobility models of the same vessel during the day and the night are also exploited to improve the prediction accuracy. Privacy issues have been taken into consideration in this paper. A quantitative model considering Markov order, training metadata and privacy leak degree is proposed to help the participant make the trade-off based on their customized requirements. We have performed extensive experiments on two years of real-world trajectory data that include more than two thousand vessels. The experiment results demonstrate that L-VTP can realize fine-grained long-term trajectory prediction with the consideration of privacy issues. The average error of 4.5-hour fine-grained prediction is less than 500 m. In addition, the proposed method can be extended to 10-hour prediction with an average error of 2.16 km, which is also far less than the communication range of ocean vessel communication devices.

## 1. Introduction

With the rapid development of IoT (Internet of Things) technology, our living environments are surrounded and monitored by sensors via IoT [1,2,3]. According to Cisco’s research report [4], 25 billion IoT devices will be connected to the Internet by 2015 and more than 50 billion by 2020, containing different kinds of marine devices. Marine IoT [5,6] has played a vital role in the domain of marine technology, showing tremendous research value for marine environmental protection, resources exploitation, etc. In many application scenarios, researchers utilize an ocean observing system to study and monitor the whole ocean, referring to ocean resources development, environmental protection and disaster warnings. For example, the GOOS (Global Ocean Observing System) [7] and U.S. IOOS (Integrated Ocean Observing System) [8] are both integrated systems for observations, modeling, and analysis of heterogeneous ocean observing data. DARPA (Defense Advanced Research Project Agency) also presents their project plans for “Ocean of Things”. However, there are several shortcomings and problems in the scenario of ocean observation: (1) limited cover of cellular signal, (2) high cost of satellite data transmission, and (3) high demand for infrastructures such as submarine optical fiber cables.

In order to build a low cost network to collect ocean data, researchers are devoted to constructing an optimal MDTN (Mobile Delay Tolerant Network) for ocean communication under a changeable and complicated environment with few wireless infrastructures [9,10,11]. If an ocean vessel’s trajectory could be obtained in advance, the MDTN delivery ratio and efficiency will be largely improved [12,13]. However, it is difficult to discover the ocean vessel’s mobility pattern for several reasons [14]. Firstly, a vessel’s mobility can be easily affected by environmental factors such as weather, tide, etc. Secondly, lacking a comprehensive tracking system to get the destination ahead of the navigation schedule. Thirdly, without any support of road topology (such as shortest path, crossroad turning possibility, etc.), an ocean vessel’s trajectory becomes unpredictable. A number of trajectory prediction algorithms have been proposed [15,16,17,18,19,20]. However, most of them are designed for pedestrian, vehicle or river vessels, which cannot be directly leveraged in the scenario of ocean vessel trajectory prediction. Others are proposed for short-term prediction within a small spatial-temporal range or regular mobility pattern. As for ocean vessel trajectory prediction, a long-term prediction algorithm outperforms a short-term one in several practical scenarios (e.g., accident warning demands hours-ahead notice for ocean vessel, especially for the large cargo vessel and tanker, which cannot speed up or slow down immediately during a short time). Moreover, even if long-term vessel trajectory prediction could be achieved, how to deal with the privacy issues is still an inevitable challenge. In our previous work [21], we simply leverage a Markov model to predict vessel trajectory without considering privacy issues, which also has an unsatisfactory performance of long-term prediction.

In this paper, we propose a long-term trajectory prediction for an ocean vessel called L-VTP. Firstly, divide the time dimension into two sub-parts in terms of vessels overall activity level. In addition, then we separately study the characteristics of vessel trajectory in different time periods. Secondly, separate a given sea area into a grid whose cell has no overlap with each other. Each cell represents a small sea area. Instead of predicting a possible location where a vessel is, we aim at getting the cell which it may be in. Thirdly, choose a vessel’s location, direction, and speed as key factors to analyze spatial-temporal regularity of vessel mobility. In terms of entropy analysis, we choose proper factors and orders of Markov chain for L-VTP. Then, calculate transition probability matrixes based on entropy results and predict vessel trajectory by using an optimized multivariate K-order Markov chain method. Finally, take privacy issues into consideration and propose a trajectory protection mechanism. In order to verify availability and efficiency of L-VTP, we carry out rigorous experiments on the two-year (from 1 January 2016 to 31 December 2017) trajectory data of two thousand vessels from the ocean and fisheries bureau of Zhejiang province. To the best of our knowledge, this is the first detailed, systematic long-term trajectory prediction strategy for ocean vessels. In addition, it is the first paper report on a personalization mobility pattern of ocean vessels with real data at this scale. We summarize the main contributions of this paper as follows:

1. We utilize MapReduce to preprocess tremendous vessel trajectory data in parallel and then leverage information entropy to analyze the effect of different vessel navigation factors and orders of Markov Chain on vessel trajectory uncertainty.
2. In terms of entropy analysis results, we utilize location, speed, and direction to calculate a transition matrix of a higher order Markov Chain, which is capable of predicting the trajectory for 4 h accurately.
3. In order to address the target missing problem, we build several transition matrices and sort them in ascending order by entropy results. When a target missing problem emerges, L-VTP automatically chooses matrices one by one in a sequence of entropy to prolong the prediction time.
4. We also take privacy issues into consideration and present a quantitative model considering Markov order, training metadata and privacy leak degree. Related practitioners can make a trade-off from among them based on their requirements.
5. We implement extensive trajectory-driven experiments, and the experimental results show that L-VTP can realize 4.5-hour precision prediction with an average error of 500 m and 10-hour prediction with an average error of 2.16 km.

The rest of this paper is organized as follows: In Section 2, a few related works about trajectory prediction are introduced. Section 3 elaborates the core of L-VTP. Section 4 verifies the utility and efficiency of L-VTP by implementing experiments with tremendous vessel trajectory data. Section 5 discusses the privacy concern about this algorithm. Finally, Section 6 concludes this paper and gives further discussion of future research work.

## 2. Related Work

Among existing related works about this paper, most of them are devoted to high accuracy trajectory prediction for pedestrians and vehicles. Song et al. predict a user’s trajectory based on 50,000 users’ base station records and utilizes information entropy results and Fano inequality to forecast a user’s mobility pattern. The average prediction accuracy is 93% [22]. Lu et al. refers to the above methods, and leverage Markov Chain to predict 500,000 user’s behavior, whose average accuracy reaches 95% [23]. However, both of the methods mentioned above did not consider other influence factors (such as time and weather) besides location. TPA [18] improves these kinds of methods. Authors separate the time into workday, weekend and holiday and divide each day into three time-slots. They build several state-transition matrices for each kind of time-day combination, which obtains more accurate prediction results. In [19], researchers predict pedestrian movement on the basis of a mixed Markov chain, taking the pedestrian’s personality and the effects of the pedestrian’s previous status into consideration. The mentioned methods are capable of predicting moving node’s behavior based on their base station or access point records. However, these methods are coarse-grained methods which cannot reach the long-term prediction purpose of ocean vessels. Zhu et al. leverage a 2-order Markov chain to predict taxies in Shenzhen and Shanghai, and verify their methods in VANETs field. However, they do not consider other factors [12].

Other researchers are working on ocean vessel trajectory prediction. Li et al. [24] leverage a multi-step clustering approach to discover regular vessel trajectory and filter irregular vessel trajectory, but lack considerations on vessel’s individual characteristics. Based on historical AIS (Automatic Identification System) data, a data-driven algorithm [25] is presented to predict a vessel’s next location, predicting well in a 30 min range. However, this approach is sensitive to the selection of certain influence factors. Lokukaluge et al. [15,16] utilize machine learning algorithms for tracking ocean vessels and then present an extended Kalman filter to predict an ocean vessel trajectory. Methods in [15,16,21,25] can only realize short-term trajectory prediction.

In order to fulfill long-term trajectory prediction for ocean vessels, we need to consider navigation factors (such as time, location, direction, speed, etc.) and construct an individual prediction model for each vessel.

## 3. Methods

**Figure 1:**
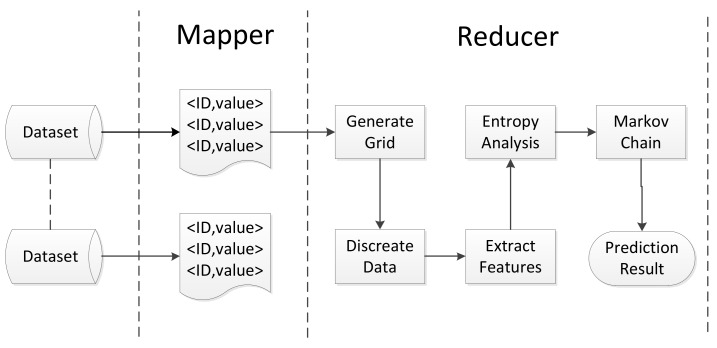
MapReduce-based architecture of L-VTP.

Based on our previous work [21], we put up with a novel algorithm to predict ocean vessel trajectory by leveraging K-order multivariate Markov Chain, and leveraged a MapReduce model to process tremendous vessel trajectory data in parallel. The MapReduce-based architecture of L-VTP is depicted in Figure 1. Based on vessel ID, L-VTP firstly utilized the Map function to generate key pairs as <vessel ID, trajectory info>, and then combined these key pairs of each vessel. Finally, we leveraged the Reduce function to fulfill vessel trajectory prediction, which contains the following three steps:

Step 1. Separate a certain sea area into a grid and each cell of the grid represents the actual trajectory location that we define and utilize in this paper.

Step 2. Based on the result of conditional and marginal entropy analysis, select vessel location, direction, and speed as influence factors to analyze spatial-temporal regularity of vessel mobility.

Step 3. Calculate transition probability matrix of K-order Markov Chain to predict vessel trajectory.

### 3.1. Mapper and Reducer

L-VTP utilizes MapReduce to preprocess tremendous vessel trajectory data in parallel. Based on the data structure of vessel trajectory, firstly leverage Map function to generate key pairs as <vessel ID, trajectory info> (see Algorithm 1), and then combine these key pairs of each vessel. Finally, adopt the Reduce function to fulfill vessel trajectory prediction (see Algorithm 2). 

**Algorithm 1:** Mapper

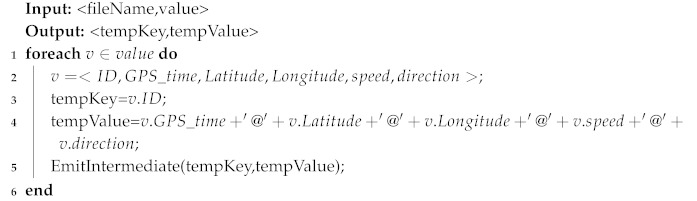



**Algorithm 2:** Reducer

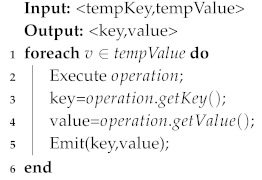



### 3.2. Generate Grid

**Figure 2:**
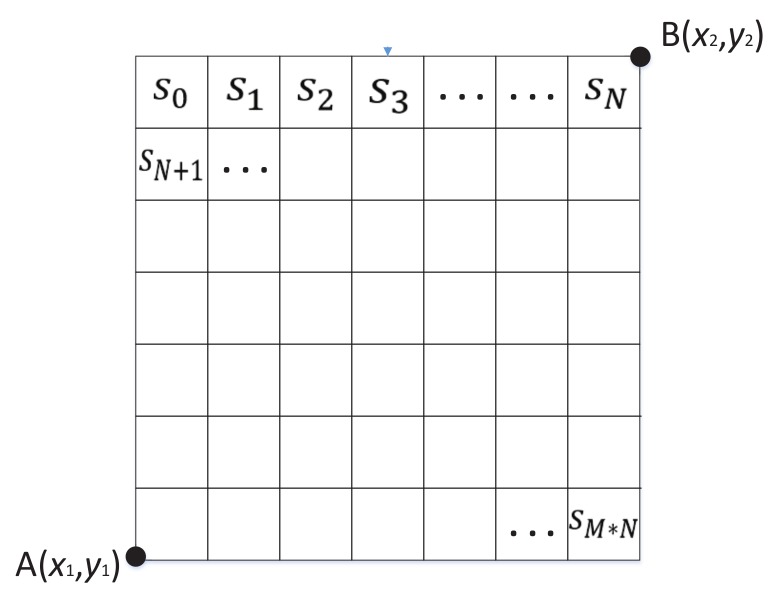
Mapping grid of sea area.

In terms of actual demand, we selected a certain sea area and separated it into an M×N grid (see Figure 2) whose cells had no overlap with each other. Each cell represents a small area of the sea. The whole sea area *S* is denoted as follows where s${}_{i}$ represents a cell:

(1) $$S=\{s_{1},s_{2},\ldots ,s_{\left(M\times N\right)}|s_{i}\cap s_{j}=\emptyset \}.$$

Grid Size: Select two points $A(x_{1},y_{1})$ and $B(x_{2},y_{2})$ to determine the grid size where the vertical direction of grid coordinate is north–south and the horizontal direction is east–west. The value of *x* denotes longitude and *y* denotes latitude.

Cell Size: $\theta_{l}$ denotes the side length of each cell and can be obtained from experiment experience. In this paper, we obtained $\theta_{l}$ by leveraging the equation $\theta_{l}=v\times t$, where *v* represents vessel’s average speed and *t* represents a sampling interval (see Algorithm 3). 

**Algorithm 3:** Generate Grid

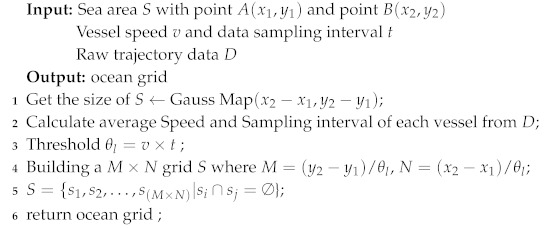



### 3.3. Data Preprocessing

s${}_{i}$ represents a small part of the sea area *S*. In each s${}_{i}$, a vessel’s mobility can be affected by the natural forces or navigation event, reflected through several factors such as speed and direction. By considering the two factors above with location, we denote a vessel trajectory characteristic λ as Equation (2):

(2) λ={Location,Speed,Direction}.

In order to implement a Markov Chain into the scenario of vessel trajectory prediction, we discreted the value of those factors by several thresholds and preprocessed the trajectory data. Moreover, the MapReduce model was adopted to improve the efficiency of preprocessing the trajectory data.

We formatted the trajectory data as key-value pairs <VesselID,TrajectoryData>, where VesselID is the unique identifier of each vessel. Trajectory data contain a vessel’s timestamp, location, direction and speed. We utilized the Map function to allocate different vessels’ data to different servers of the cluster. The data with same key will be put on the same server. Then, the reduce function was leveraged to realize the data preprocessing algorithm. The whole data preprocessing process is detailed in Algorithm 4. 

**Algorithm 4:** Data Preprocessing

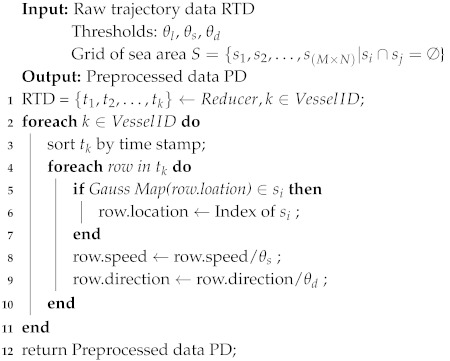



### 3.4. Entropy Analysis

In terms of information theory, entropy analysis is a mathematical approach of measuring the disorder degree. The entropy could represent the uncertainty of vessel trajectory to some extent. In this paper, the unpredictability of the trajectory dataset, with greater randomness implying high entropy and greater predictability implying lower entropy. Moreover, conditional entropy represents the probability of prediction ahead of knowing the previous status. The result of entropy analysis will determine how we process our data (i.e., use the latest k state to prediction the next one) before flushing it into the Markov model. Theoretically, the order of conditional entropy is unlimited. We can utilize a higher order of Markov to get better prediction performance while a higher order will bring more computation overhead. In order to achieve high prediction precision with low computation overhead, we leveraged an entropy analysis method to choose an appropriate order of the Markov chain.

After data preprocessing, each vessel’s data will be allocated to the same server. Thus, we utilized a parallel approach to calculate entropy from trajectory big data. We select the order of a Markov Chain and the key factors for vessel trajectory prediction by leveraging entropy analysis (see Algorithm 5) as follows:

Step 1. Obtaining the preprocessed data through Algorithm 4, we assume that $vessel_{i}$ has a trace $t_{i}$ denoted in Equation (3), where λ is defined in Equation (2). *M* represents the number of time slots.

(3) $$t_{i}=\left\{\lambda_{0},\lambda_{1},\ldots ,\lambda_{\left(M-1\right)}\right\}.$$

Step 2. For all the $\lambda_{j}$ of $t_{i}$, we count how many times $\lambda_{j}$ occurs and denote it as $m_{\lambda_{j}}$, where 0≤j≤(L×S×D−1):

- *L* = Max($\lambda_{j}$.location),
- *S* = Max($\lambda_{j}$.speed),
- *D* = Max($\lambda_{j}$.direction).

Step 3. In terms of frequency($m_{\lambda_{j}}/M$), we calculate the marginal entropy of $t_{i}$ (see Equation (4)) and extend $t_{i}$ to a sequence of two-tuples ${t_{i}}^{1}$ = {$\left(\lambda_{0},\lambda_{1}\right),\left(\lambda_{1},\lambda_{2}\right),\ldots ,\left(\lambda_{M-2},\lambda_{M-1}\right)$}, count how many times $\left(\lambda_{\phi },\lambda_{\xi }\right)$ occurs in ${t_{i}}^{1}$ and denote it as $m_{\phi ,\xi }$, finally get joint $H({t_{i}}^{1},t_{i})$ entropy as Equation (5):

(4) $$H\left(t_{i}\right)=\sum_{j=0}^{L\times S\times D-1}\frac{m_{\lambda_{j}}}{M}\times \log_{2}\frac{1}{m_{\lambda_{j}}/M},$$

(5) $$\begin{array}{c} H\left(t_{i}^{1}|t_{i}\right)=\sum_{\forall 0\leq \phi ,\xi \leq (L\times S\times D-1)}\frac{m_{\phi ,\xi }}{M-1}\times \log_{2}\frac{1}{m_{\phi ,\xi }/\left(M-1\right)}. \end{array}$$

Step 4. Calculate conditional entropy of ${t_{i}}^{1}$ by utilizing Equation (6):

(6) $$H\left(t_{i}^{1}|t_{i}\right)=H\left(t_{i}^{1},t_{i}\right)-H\left(t_{i}\right).$$

Step 5. Based on Step 1–Step 4, keep calculating the conditional entropy of ${t_{i}}^{k}$ and eventually the conditional entropy of ${t_{i}}^{k}$ is written as Equation (7):

(7) $$\begin{array}{c} H\left(t_{i}^{k}|t_{i}t_{i}^{1}\ldots t_{i}^{k-1}\right)=H\left(t_{i}^{k},t_{i}t_{i}^{1}\ldots t_{i}^{k-1}\right)-\\ H\left(t_{i}^{k},t_{i}t_{i}^{1}\ldots t_{i}^{k-2}\right)-\ldots H\left(t_{i}\right). \end{array}$$

**Figure 3:**
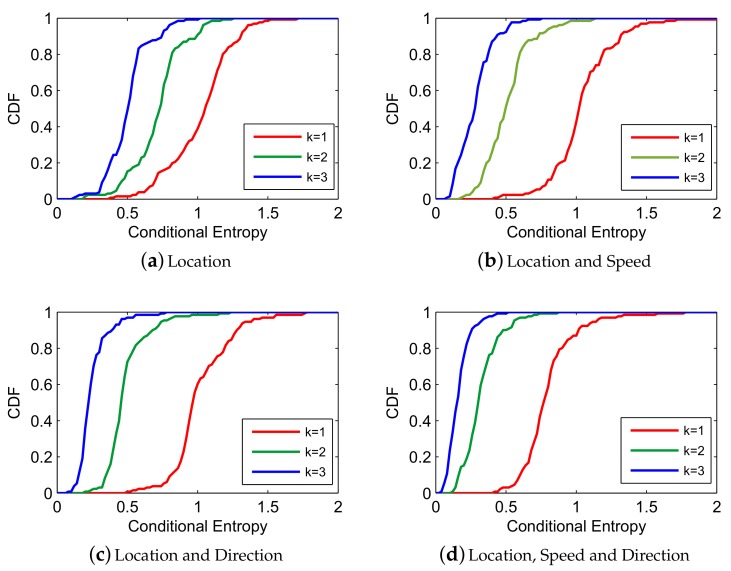
Conditional entropy comparison between different *k* with fixed factors [21].

**Figure 4:**
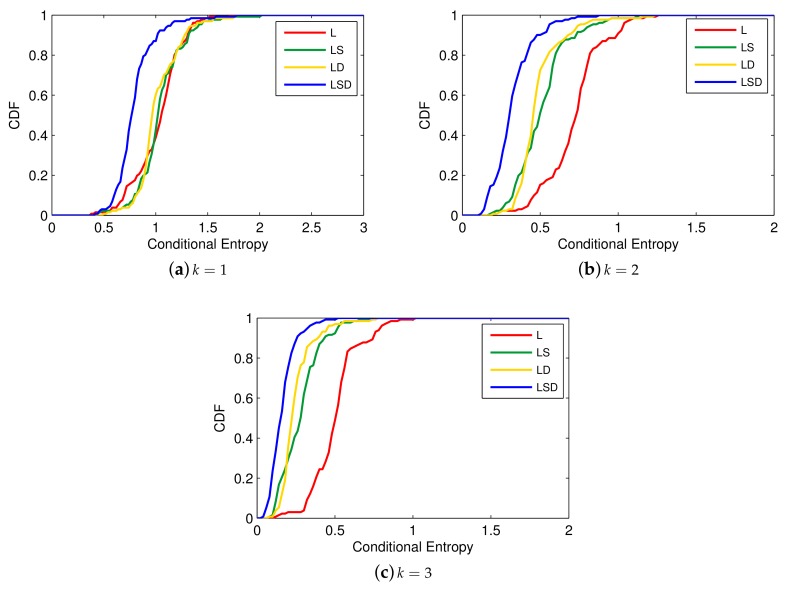
Conditional entropy comparison between different factors with fixed *k* [21].

In order to select the order of Markov Chain and key factors of vessel trajectory, we carry out a few experiments under each possible situations, shown in Figure 3 and Figure 4 (*L* denotes Location, *S* denotes Speed, *D* denotes Direction), where a higher level of conditional entropy represents less uncertainty of vessel trajectory prediction. Figure 3 reveals that 3-order conditional entropy performs better than 1-order and 2-order conditional entropy with different factors. Moreover, with the increasing number of factors, the performance of conditional entropy between 2-order and 3-order becomes closer, but both are getting further from 1-order entropy. Figure 4 depicts the effect of selecting key factors on trajectory prediction. Obviously, utilizing all the factors (location, speed, direction) with 3-order conditional entropy performs optimally. In Figure 4a, a slight difference emerges when utilizing different factors, and it is shown in Figure 4b,c that (location, direction) works better than (location, speed) with higher order conditional entropy. In a word, a higher accuracy prediction result can be obtained by utilizing higher-order conditional entropy with multiple factors. In this paper, we end up with 3-order due to the continuous growth of spatial-temporal complexity for L-VTP. 

**Algorithm 5:** Conditional Entropy Calculation

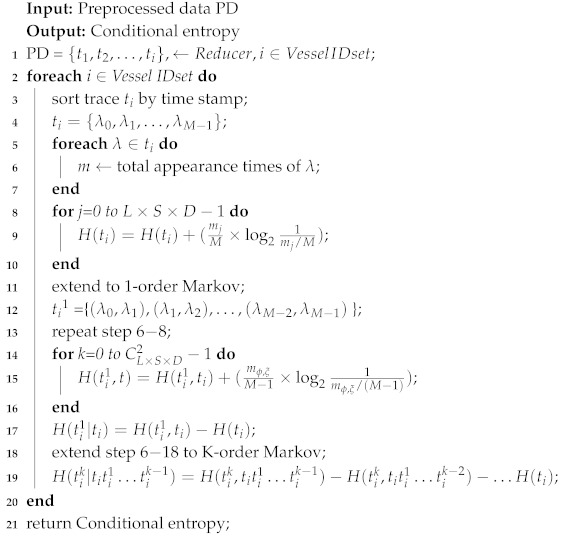



### 3.5. Vessel Trajectory Prediction

Given a K-order Markov Chain, we calculate the transition probability matrix *P* of each vessel with different time divisions. *P* denotes vessels moving pattern under different situations, defined as the following equation:

(8) $$P=\left[\begin{array}{ccc} p_{c^{1},e^{1}} & \ldots & p_{c^{1},e^{L\times S\times D}}\\ ⁝ & \ddots & ⁝\\ p_{c^{{\left(L\times S\times D\right)}^{k}},e^{1}} & \ldots & p_{c^{{\left(L\times S\times D\right)}^{k}},e^{L\times S\times D}} \end{array}\right].$$

*c* represents vessel current *k*-state, *e* represents the next state. $p_{c^{i},e^{j}}=M\left(e^{j}|c^{i}\right)/M\left(c^{i}\right)$, $M(c^{i}$) represents the frequency of state $c^{i}$, and $M(e^{j}|c^{i})$ represents the frequency of current *k*-state $c^{i}$ with the next state $e^{j}$.

More precise prediction can be obtained by leveraging higher-order Markov with multiple factors. Although we get a transition probability matrix after calculating massive training data, there still exists a probability that a new state will emerge and can’t be found in transition probability matrix, leading to a blackout of the vessel trajectory prediction process. To address the problem, we propose an optimizing algorithm based on the K-order Markov shifting method. Once a new state appears and we can’t find any matching in current transition probability matrix, our algorithm keeps shifting K-order Markov to a lower (K-1)-order Markov automatically until a matching is found in the relative transition probability matrix. We utilized the lower-order Markov matching result to reconstruct the previous state and enabled the prediction process to continue from the blackout. The details are shown in Algorithm 6 and Table 1.

**Algorithm 6:** Prediction Optimization

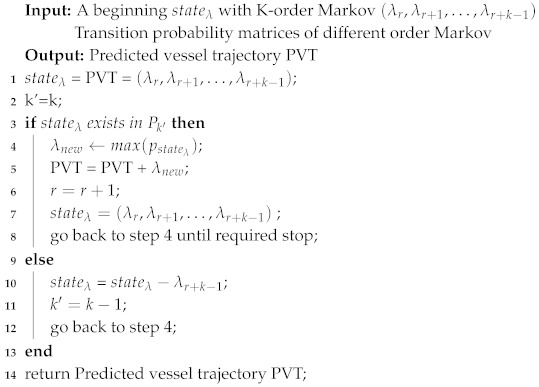



## 4. Experiments and Discussion

In this section, we implement extensive trajectory-driven experiments by utilizing tremendous fishing vessel trajectory data. Based on the experiments’ analysis results, we demonstrate the utility and performance of L-VTP.

### 4.1. Experiments’ Setup

The experiment dataset is provided by the security and emergency center of the Wenzhou oceanic and fishery administration in China. In order to verify the performance of L-VTP, we utilize more than 300 GB BDS (BeiDou Navigation Satellite System) records from 1 January 2016 to 31 December 2017, including 2116 fishing vessels. In this dataset, vessel trajectory is composed of time series data, and each record contains the information of vessel ID, GPS_time, latitude, longitude, speed and direction (see Table 2). This trajectory dataset can be leveraged in other scenarios, such as ocean MDTN, vessel mobility pattern mining, and fishery management.

**Figure 5:**
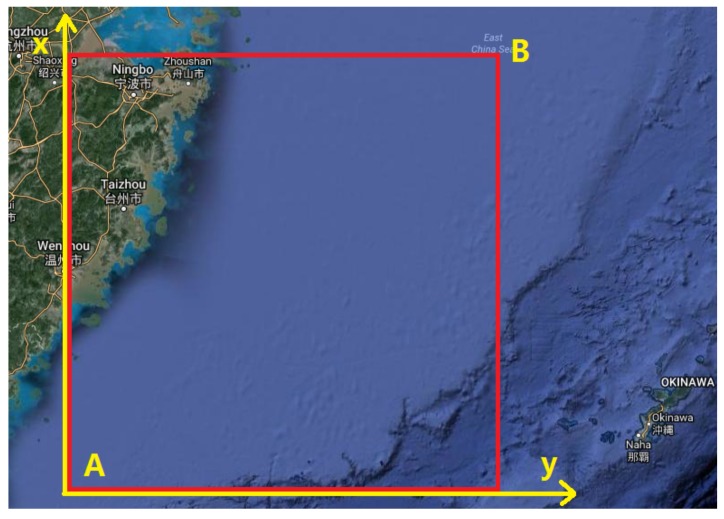
Chosen sea area with relative coordinates.

We select a sea area in the East China Sea, which is shown in Figure 5. Based on the Beijing Geodetic Coordinate System (BJZ54), we implement coordinate transformation by utilizing a Gaussian Map, from GPS geodetic coordinates into Gauss geodetic coordinates, and then construct L-VTP coordinates by leveraging point A (25.616141${}^{{}^{\circ}}$ N, 120.692423${}^{{}^{\circ}}$ E) in the bottom-left corner as the coordinate origin and point B (30.377660${}^{{}^{\circ}}$ N, 125.894656${}^{{}^{\circ}}$ E) to determine the grid size. We select the vertical direction as the *x*-axis pointing North and the horizontal direction as the *y*-axis pointing East. In the end, taking vessel average speed and sampling interval into consideration, we set the cell length $\theta_{l}$ = 1 km and present the setup for other basic parameters in Table 3.

### 4.2. Results and Discussion

**Figure 6:**
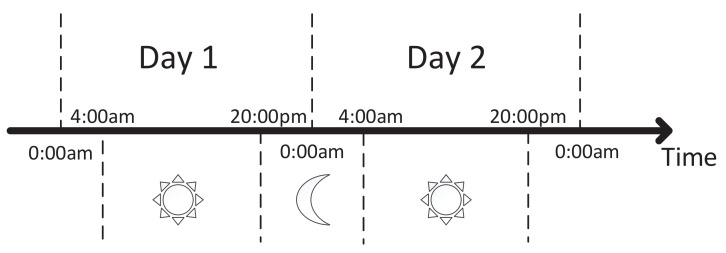
Time division by day and night.

A vessel’s mobility pattern is closely related to the time of the day because of the special sailing behavior of vessels in the sea. For instance, a vessel sails faster and changes direction with a larger angle more frequently in the day compared to in the night. In addition, the vessels are more likely to sail away from the seashore in the daytime. Therefore, taking the time into consideration will make contributions to vessel trajectory prediction. There are many meaningful ways to separate time according to the fish moratorium, tide, weather, etc. In our previous research [14,26], a parallel algorithm is proposed to detect the spatial-temporal contact pattern from trajectories of over 2000 vessels. Based on quantity analysis of inter-contact time distribution, contact times distribution, sailing time distribution, etc., we divide 24 h into two parts: day and night, including 4:00 a.m.–8:00 p.m. and 8:00 p.m.–4:00 a.m., shown in Figure 6. Once the trajectory pattern is divided into sub-parts by time dimension, we separately study the characteristics of the vessel trajectory in different time periods. In terms of entropy analysis results from Section 3.4, we choose (location, speed, direction) as key factors to continue the following experiment and select thresholds.

Markov Chain leverages previous states with the maximum probability to predict the next state, making location prediction at each different time very important. However, the maximum probability method cannot ensure the right prediction result. Limited by the amount of trajectory data, we respectively choose three groups of 3-month, 6-month, 9-month and 12-month trajectory data as training data, and the rest of them as test data, in order to calculate the error ratio of the Markov choosing maximum probability state at each different time. As shown in Table 4, the average error ratio of Markov decreases severely as we utilize more training data. Meanwhile, the standard deviation also increases, revealing that the vessel moving pattern varies with seasonality.

**Figure 7:**
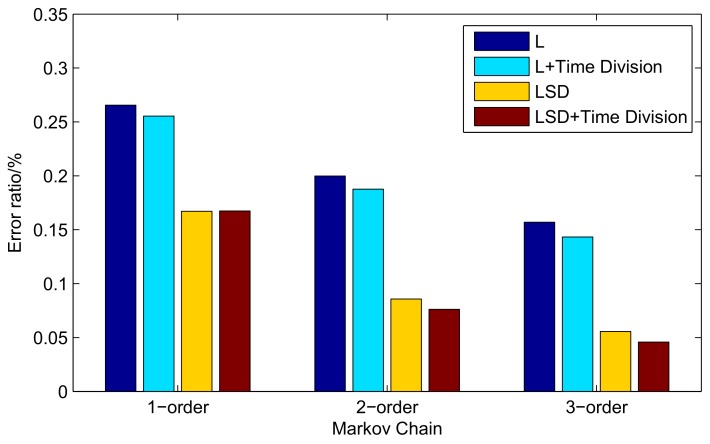
Error ratio of the Markov choosing maximum probability state at each different time.

Therefore, we utilize the 2016 dataset to train the matrix and the data of 2017 as test data in the following experiments. As shown in Figure 7, there is a 17% error ratio when using 1-order Markov. However, with the higher order of Markov, the error ratio decreases by 50% from 1-order Markov to 2-order Markov and even more than 70% to 3-order Markov. In addition, time division makes a further contribution to improving the success ratio.

**Figure 8:**
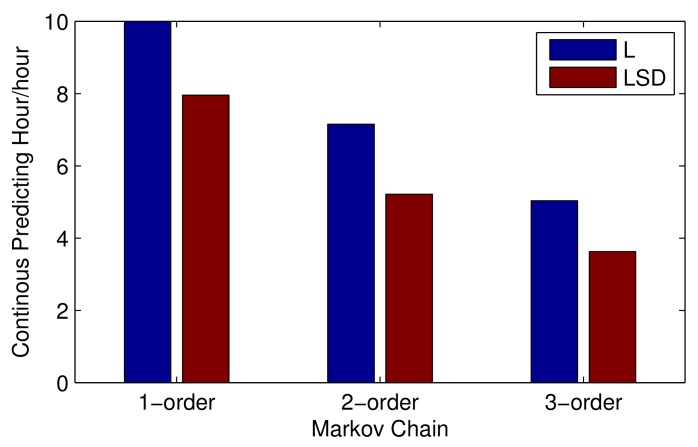
Continuous predicting hours under different orders of Markov.

There exists a probability that a new state will emerge and can not be found in the transition probability matrix, leading to a blackout of the vessel trajectory prediction process, especially when using higher-order Markov with multiple factors. As shown in Figure 8, putting prediction accuracy aside, a 1-order Markov with factor location is able to keep predicting for more than 10 h. After taking speed and direction into account, the continuous predicting hour drops to 8 h on average. With the increasing order of Markov, less predicting hours have been obtained and, finally, the 3-order Markov with (location, speed, direction) can only predict for less than 4 h. Therefore, we propose a K-order Markov shifting method to solve this problem.

**Figure 9:**
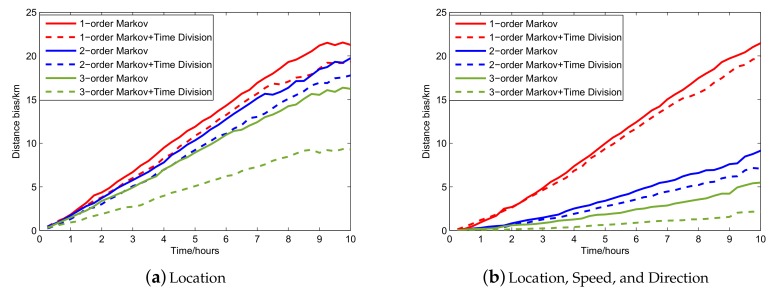
Variation tendency of bias error as time increases.

**Figure 10:**
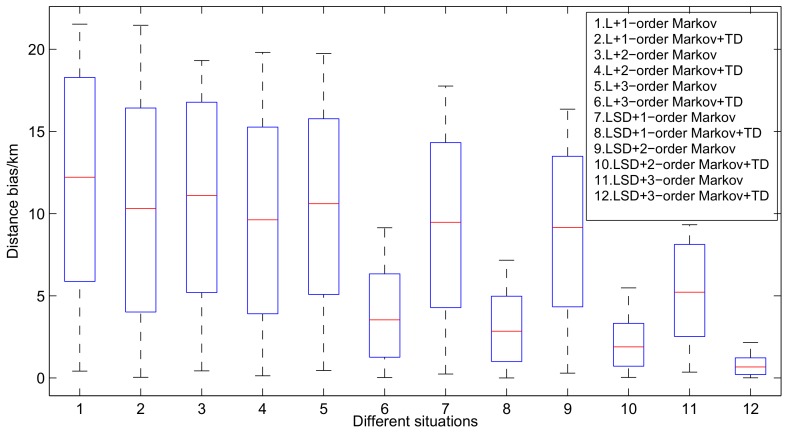
Variation tendency of bias error under different circumstances.

**Figure 11:**
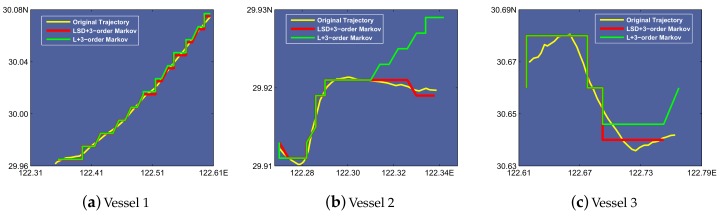
Comparison between original trajectory and predicted trajectory under 3-order Markov with different factors.

**Figure 12:**
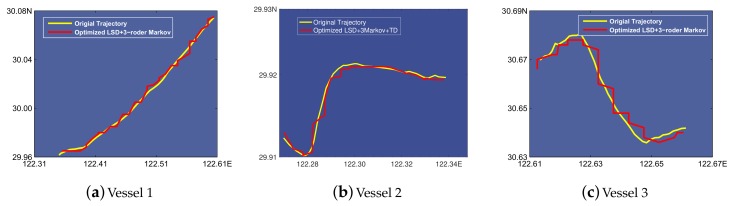
Comparison between original trajectory and predicted trajectory under 3-order Markov with factor Location after optimized.

Based on the results depicted in Figure 8, we select the starting points of prediction every 10 h and leverage a 10-hour range of vessel trajectory data to demonstrate the L-VTP’s utility for short- and long-term period prediction. Figure 9 depicts the variation tendency of bias error as time increases. After using the K-order Markov shifting method, no blackout occurs and we can get a complete prediction trajectory in the end. Obviously, a higher order improved Markov with temporal division has a much better performance at short and long-term period prediction. Although predicted location gets farther from the actual location, there is nearly a 100% success ratio of prediction until four hours pass, and bias distance is controlled under 2 km in a 10-hour trajectory. In Figure 10, we can see that 3-order improved Markov with temporal division has the best performance and increases precision by 50% for vessel trajectory prediction. Finally, we choose three vessels and compare their original trajectory with the predicted trajectory (see Figure 11). As described in Section 3.2, we leverage the center of the belonging cell as the predicted location, which can be optimized. Although we represent the vessel’s location in cell granularity, sometimes it is incorrect to choose the center of the cell as a drawing point in our map. Thus, we optimize a vessel’s location mainly based on its last-reported direction and also take the last drawing point into account. For example, assuming the location of the last point is in the center, if we find that the vessel’s last-reported direction is less than 45${}^{{}^{\circ}}$, we may choose the midpoint of the cell’s right boundary instead of the center of it. The optimized results are shown in Figure 12.

## 5. Privacy Concern

In the Ocean MDTN scenario, L-VTP is proposed to exchange their mobility pattern (transition probability matrix) and improve the message transmission efficiency among the networks. However, the efficient long-term trajectory prediction algorithm results in a privacy leak problem. In this section, we take privacy issues into account and give a quantitative model considering Markov order, training metadata and privacy leak degree. The definition of Privacy Leak Degree is given below.

**Privacy Leak Degree**: Privacy Leak Degree is defined as the maximum continuous time (hours) under a certain tolerant distance bias.

For example, if the communication radius is 1 km, we assume that the participant among the networks can tolerate the distance bias of 1 km. In Figure 9, we can see that the maximum continuous time is about one hour under a location only 3-order Markov chain, so the Privacy Leak Degree of a location only 3-order Markov chain is one that is under a 1 km tolerant distance bias.

**Figure 13:**
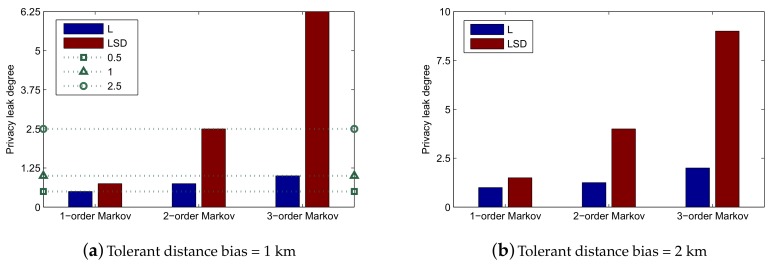
Privacy leak degree under different tolerant distance bias.

Figure 13 shows the privacy leak degree under different tolerant distance bias situations. In Figure 13a, we can see that the privacy leak degree of 3-order Markov LSD can reach about 6.25, which means that its continuous prediction time is 6.25 h with the distance bias under 1 km. The privacy leak degree of 2-order Markov LSD can reach 2.5.

If the participant is a delay sensitive user, which means he/she wants to receive the message in time, he/she needs to sacrifice his/her privacy to get better network quality. Otherwise, if the participant is an announcer, he/she could select a method with a low privacy leak degree.

In this section, we propose a method to help the participant make a trade-off from among Markov order, training metadata and privacy leak degree. The algorithm needs to select the most accurate prediction method and follow the privacy requirements and tolerant prediction bias. From Figure 9, we observe that prediction time and distance bias obey linear function, and we could approximate 12 linear functions using the least squares method. The function is as follows:

(9) $$bias=\eta_{\gamma }PLD,$$

where γ stands for each method of L-VTP ranging from 1 to 12. γ follows the descending sequence of slope. PLD stands for Privacy Leak Degree. First, the participant will select a prediction bias β and PLD τ he/she can tolerate. Then, he/she will traverse and calculate PLD by the order of γ. The participant should select the method that has less PLD but is the closest to τ. The details of the algorithm are shown as Algorithm 7. 

**Algorithm 7:** Method Selection

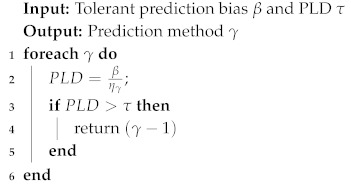



## 6. Conclusions and Future Work

In this paper, we propose a long-term trajectory prediction for an ocean vessel called L-VTP by leveraging a K-order multivariate Markov Chain. Firstly, we utilize MapReduce to preprocess tremendous vessel trajectory data in parallel. Secondly, we separate a certain ocean area into a grid through coordinate transformation. Thirdly, in terms of entropy analysis results, we select (location, direction, speed) as the key factors of the vessel’s trajectory data and the optimal order of Markov Chain to analyze spatial-temporal regularity of vessel mobility. Finally, we calculate transition probability matrix and predict vessel trajectory by using the K-order Markov method. In addition, we take the privacy problem into consideration and present a method to let the participant have a trade-off from among Markov order, training metadata and privacy leak degree. For future work, we will apply L-VTP to solve ocean MDTN problems. Furthermore, we will research the trajectory privacy protection mechanism in the MDTN field. 

## Figures and Tables

**Table 1:** Conditional Entropy.

No	K-Order	Factor	Min/Max	Average (Desc)	Standard Deviation
1	1	L, S	(0.4105, 2.0128)	1.0426	0.2255
2	1	L	(0.3774, 1.7155)	1.0218	0.2285
3	1	L, D	(0.4975, 1.7664)	1.0179	0.2064
4	1	L, S, D	(0.4019, 1.7752)	0.7955	0.1931
5	2	L	(0.1836, 1.2428)	0.7151	0.1862
6	2	L, S	(0.1726, 1.1207)	0.508	0.1688
7	3	L	(0.1082, 1.0036)	0.5034	0.1487
8	2	L, D	(0.1815, 1.2258)	0.4849	0.1492
9	2	L, S, D	(0.1044, 0.8763)	0.3213	0.13
10	3	L, S	(0.073, 0.7411)	0.2788	0.1248
11	3	L, D	(0.078, 0.7654)	0.2489	0.1061
12	3	L, S, D	(0.0379, 0.5047)	0.1625	0.0816

**Table 2:** The format of trajectory data.

Vessel ID	GPS_Time (s)	Latitude (${}^{{}^{\circ}}$)	Longitude (${}^{{}^{\circ}}$)	Speed (nm/h)	Direction (${}^{{}^{\circ}}$)
29,229	1,420,041,972	28.8401154	122.316762	7.1	174
65,644	1,420,041,786	29.9443883	123.483638	1.1	48
… …	… …	… …	… …	… …	… …

**Table 3:** Experiment setup for basic parameters.

Parameter	Threshold	Theshold Value
Location	$\theta_{l}$	1 km
Speed	$\theta_{s}$	1 nm/h
Direction	$\theta_{d}$	90${}^{{}^{\circ}}$
Sampling Interval	t	30 min

**Table 4:** The error ratio of the three groups of training data of the Markov choosing maximum probability state at each different time.

Training Data Size	Average (%)	Standard Deviation (%)
3 months	67	4.1568
6 months	44	3.5977
9 months	18	2.0102
12 months	7	1.9996
